# Motivational Factors Affecting Iranian English as a Foreign Language (EFL) Learners’ Learning of English Across Differing Levels of Language Proficiency

**DOI:** 10.3389/fpsyg.2022.869599

**Published:** 2022-04-12

**Authors:** Reza Bagheri Nevisi, Ala Farhani

**Affiliations:** Department of English Language and Literature, University of Qom, Qom, Iran

**Keywords:** motivational factors, proficiency level, learning English, EFL learners, intrinsic motivation, extrinsic motivation

## Abstract

The present study aimed at investigating the motivational factors affecting Iranian learners’ learning of English as a Foreign Language across differing levels of language proficiency. To this end, 110 males and 70 females with an age range of 18–31 took part in the study and a mixed-methods approach was adopted. First, the researchers administered Oxford Placement Test (OPT) to determine the proficiency level of the participants and placed them into three levels of language proficiency. Elementary, intermediate, and advanced. Next, as for the quantitative part of the study, the Attitude/Motivation Test Battery (AMBT) questionnaire was distributed among all the participants to delve into the motivational factors. Finally, a semi-structured interview was conducted to qualitatively probe into EFL learners’ motivational factors affecting their learning of English. It was found that the motivational factors which encouraged EFL learners resulted from either intrinsic or extrinsic tendencies. The recognized intrinsically-based motivational factors were personal enjoyment, social prestige, and being challenged. The extrinsically-oriented factors as the source of motivation to learn English were getting a job, pursuing education, earning more money, traveling to foreign countries, pursuing academic careers, making efficient use of technology, and effectively interacting with native speakers. It was concluded that beginner and intermediate English language learners tended to learn English more based on extrinsic factors whereas advanced learners were found to be more intrinsically motivated.

## Introduction

Motivation plays a pivotal role in the psychological and pedagogical settings. However, attempts to theorize motivational factors through a single mechanism have failed and are mainly done away with (Dörnyei, 2003; Lifrieri, 2005). Nevertheless, the last two decades have seen a re-emergence and revival of interest in motivational factors, and a resurgence in intricate analyses of motivation.

Motivational factors affect the choice of language learning strategies such as memory, socio-affective, and cognitive ones (Gardner and Lambert, 1972; Dörnyei, 2005; Tabatabaei and Molavi, 2012; Zarei and Elekaei, 2013). Zheng et al. (2019) examined the relation between motivation and language proficiency and asserted that integrative and instrumental motivations enhanced learners’ foreign language proficiency and stated that this was made possible through foreign language enjoyment. As far levels of proficiency of learners are concerned, motivational factors have received insufficient attention.

Although, motivation and some of its aspects has been investigated abundantly in the literature (e.g., Kiany et al., 2013; Haji Mohammadi, 2017; Namaziandost et al., 2019), the motivational factors across varying proficiency levels that support and sustain the learning process have not been specifically dealt with. Accordingly, this study adopted a mixed-method approach and intended to delve into the motivational factors affecting EFL learners English learning across different levels of language proficiency both qualitatively and qualitatively.

## Literature Review

### Motivation Theories

From the early beginning of the 20th century, motivation has been abundantly dealt with in the literature. Despite developing many theories and research, motivation is still a controversial topic and remains a bone of contention among many researchers and practitioners within the field of applied linguistics. Many scholars used the most known theories and models of motivation as a starting point for their work within the realm of motivation. Armstrong (2007) summarized those theories in his book about employee reward management. He argued that Taylor’s theory of motivation to work is related to compensations and penalties which are directly connected to performance. A less instrumental approach is Maslow’s concept of the hierarchy of needs which describes motivation emanating from people’s unsatisfied needs. The distinction between extrinsic and intrinsic motivators was the study of Herzberg. Those “old” theories are important but not perfect (Liando et al., 2005).

#### Content Theories

Emphasizing what motivates people, requirements that are the same for everyone. Despite assuming a similar set of needs for all people, they define the needs and requirements of everyone differently. Maslow’s theory about human needs is the most well-known in the field of motivation. He states that human behavior is driven by the existence of unsatisfied needs (McClelland, 1953).

#### Process Theories

A dynamic character characterizes the process theories, not static as content theories. What motivates people is the main concern, not how motivation occurs. Process theories explain the way of behavior and attitudes of people which are aimed at certain options. Process theories focus on individual’s mental processes in specifying the motivational level (Fincham and Rhodes, 2005).

#### Self-Determination Theory

Self-determination theory (SDT) is one of the most all-embracing and inclusive motivational theories, the primary tenets of SDT are explained and delineated by Ryan and Deci (2000). Three fundamental human needs are put forward in SDT that are necessary for the general welfare and beneficial advancement of human beings: Competence, autonomy, and relatedness. Competence refers to comprehending one’s efficacy in practicing the right skills; autonomy refers to understanding yourself as the source of action, and relatedness refers to a sense of security and belonging to others. SDT states that human beings act efficiently when societal and contextual elements reinforce and sustain the gratification of the three fundamental needs. In contrast, when the circumstances do not maintain and sustain the fulfillment of essential needs, individuals display lower incentives. The three constructs were widely experimented with and were shown to influence both intrinsically and extrinsically motivated behaviors (Ryan and Deci, 2000; Guilliateaux and Dornyei, 2008).

### Empirical Studies

Kiany et al. (2013) examined motivational developments of learners in a traditional context of English education. The study investigated the combined impacts of these contextual features on learners’ motivational developments after 4 years of learning English at high schools. The data was gathered through administration of a valid and reliable questionnaire that measured the “L2 Motivational Self System” Approximately 400 Iranian high school students attended the study. Findings indicated that all the motivational factors decreased during the senior year of high school English language teaching and only “L2 anxiety” grew. The decrease in “interest,” “ideal L2 self,” and “instrumental-promotion” were significant. The study concluded that these developments in learners’ motivation were caused by traditional teaching and learning environment and conventional or die-hard policies of foreign language teaching. The study further implied that boosting learner motivation needs scientific reforms within the educational system.

Setiyadi and Sukirlan (2016) carried out a research to specify the motivational orientations of EFL university students in Indonesia and explored whether the Western motivational theories were still pertinent to them. The study included 886 university learners. The findings revealed that three motivational orientations existed among EFL learners in Indonesia: Extrinsic, international, and intrinsic orientation.

Haji Mohammadi (2017) investigated Iranian EFL learners’ L2 motivational self-system. The study sought to ascertain the possible associations between two facets of L2 selves (i.e., ideal L2 self and ought-to L2 self) and perceptions about L2 culture and community. To this end, a large-scale attitudinal questionnaire by Taghuchi et al. (2009) was distributed among 120 Iranian EFL learners. The findings corroborated the fact that both ideal and ought-to L2 selves were highly correlated with perceptions about L2 culture and community. Nevertheless, the degree of correlation of ideal L2-self seemed to be much stronger than that of ought-to L2 self. Furthermore, results showed that L2 Iranian EFL students strongly associated with L2 culture and their selves, specifically their idealized self-image, were influenced and molded by L2 cultural values. In the same vein, their obliged self-image was impacted by societal values.

Gamlo (2019) investigated the impact of combining mobile-game-based language learning applications on Saudi female EFL learners’ motivation to learn English. Thirty beginners took part in the study. The study was conducted over a seven-week period. Data were gathered via two different questionnaires. The findings of the pre-MGBLLAs combination indicated that the Saudi learners were motivated to learn English. Nevertheless, they were instrumentally motivated, since English was taught as a mandatory course and they had to attain high scores to be able to embark upon studying their preferred field.

Abdullah et al. (2019) explored the impact of carrying out the Flipped Classroom Model (FCM) on the motivational level of Omani EFL learners. A mixed-methods design was adopted. In the quantitative phase, a Motivation in English Speaking Performance Questionnaire was given out in three stages: before, during, and after the implementation of FCM over 15 weeks. Moreover, in the qualitative phase, reflective journal forms were amassed to glean more detailed information. The results revealed that applying FCM as a pedagogical tool enabled educators to create an inventive, charming, and inspiring atmosphere in the EFL speaking class. Abdullah et al. (2019) explored the impact of carrying out the FCM. The study also implied that FCM made considerable contributions to boosting pupils’ motivation in English speaking performance over time.

Namaziandost et al. (2019) examined the effect of cooperative learning on promoting Iranian intermediate EFL students’ oral proficiency motivation. To this end, 90 students were selected and further subdivided into two equal groups; one experimental and one control group. Findings were indicative of the fact that the learners’ overall oral proficiency level significantly improved after being exposed to cooperative learning instructional techniques. Moreover, findings demonstrated significant differences in support of cooperative learning to enhance intrinsic motivation, but no significant differences were seen in other kinds of motivation.

Wallace and Leong (2020) probed into motivational factors that affected young EFL learners. More specifically, it delved into their perceptions and objectives to study English, and how their perceived notions of societal support and that of the formal learning milieu (pedagogical activities and classroom tasks) ranged at differing motivational levels. A total number of 23 six-graders filled out a 10-item open-ended questionnaire. Results demonstrated that majority of students were highly motivated and learned English for both integrative and instrumental purposes.

Going through the related literature, it seems that there exists a paucity of research dealing with motivational factors that affect EFL learners’ learning of English in the Iranian context at differing levels of language proficiency. More specifically, most of the previously-done studies have adopted either a quantitative approach or in few instances a qualitative approach separately without adopting a combination of both to delve into the motivational factors. Moreover, not only did the study intend to adopt a mixed-methods design to augment the overall validity and reliability of research findings, but also it went even further to see whether any discernible pattern of motivational factors existed for EFL learners across differing levels of language proficiency. Therefore, the researchers formulated the following research questions to meet the above-stated objective of the research:

(1) What motivational factors influence Iranian EFL learners’ English language learning?
(2) What motivational factors influence the Iranian EFL learners’ English language learning across different English proficiency levels (elementary, intermediate, and advanced)?
(3) Are there any common motivational themes among Iranian learners at different levels of language proficiency?
(4) Is there any significant difference among Iranian elementary, intermediate, and advanced EFL learners in terms of the motivational factors which influence their English learning?

## Methodology

### Participants

Two-hundred Iranian EFL learners studying English in Gooyesh, Rohroshd, and Safir Language Institutes in Qom, Iran took part in the study. Due to the special conditions imposed by the COVID-19 pandemic, from among this number, 180 EFL learners (110 males and 70 females) from different language proficiency levels (elementary, intermediate, and advanced) could complete the entire course of the study. Their age ranged from 18 to 31. The demographic information of the participants is presented in Table 1.

**TABLE 1:** Demographic features of the participants.

Demographic features	Categories	*N*	Percentage (%)
Gender	Male	110	61
	Female	70	49
Language proficiency	Elementary	82	45.5
	Intermediate	70	39
	Advanced	28	4.5
Age	Under 18	59	25.5
	18–24	67	37.2
	24–31	54	31.1

### Instruments

#### Oxford Placement Test

To determine the participants’ language proficiency level, the researchers utilized Oxford Placement Test (OPT) at the beginning of the study. Also, using the KR21 formula, the internal consistency of the test was calculated and reported (*r* = 0.86), which was fairly satisfactory. Furthermore, to ensure its validity, it was reviewed by two language experts and their comments were applied in the follow-up version of the main study. The OPT consisted of three parts: Vocabulary Test, Reading Comprehension, and Grammatical Structures. This test included 40 vocabulary tests, 40 grammar tests, and 20 reading comprehension tests.

#### Semi-Structured Interview

To collect the data for the qualitative part of the study, the researchers conducted a semi-structured interview to delve into the students’ self-report perceptions of the motivational factors which might have affected their English learning process. The interview questions were developed by the researchers. To ensure the validity of the questions, the researchers handed items over to three professors of Teaching English as a Foreign language (TEFL): One full professor and two associate professors. They were kindly asked to go through the related questions and determine whether they were appropriate based on the participants’ proficiency level and the purpose of the study. Accordingly, a pilot study was conducted before the main study to probe into the potential drawbacks of the questions.

#### Questionnaire of EFL Learners’ Motivational Factors

The questionnaire was adopted from Gardner’s Attitudes/Motivation Test Battery (AMTB). This questionnaire is composed of 10 multiple-choice items on a 5-point Likert scale. Furthermore, to make sure about the reliability of this research instrument, the researchers conducted a pilot study with 30 participants before starting the main phase of the study and the obtained coefficient was found to be 0.79 which is considered an acceptable and satisfactory level of internal consistency.

### Data Collection Procedure

The following steps were taken to achieve the already-stated objectives of the study. First, the researchers administered OPT to determine the proficiency level of 200 participants and placed them into three levels of language proficiency: Elementary, intermediate, and advanced. Next, as for the quantitative part of the study, the researchers distributed the AMBT questionnaire among all the participants to delve into the motivational factors that could have affected their English learning. Finally, a semi-structured interview was conducted to qualitatively probe into EFL learners’ motivational factors affecting their learning of English.

### Coding Procedure

Having recorded all EFL learners’ responses to the semi-structured interview questions, the researchers went through the recorded data as thoroughly and meticulously as possible and transcribed the recordings to determine the most typical themes within the transcriptions. The thematic analysis was done based on Braun and Clarke (2006). To achieve coding, the two researchers went through the EFL learners’ transcribed responses to the questions of the semi-structured interview one by one and decided upon the most typical motivational themes that could be extracted. It turned out that the two coders almost concurred with one another in most cases and the intercoder reliability stood at 0.76. However, the two coders turned to a writing expert for help in case of discrepancies to make more informed decisions. The expert enjoyed 25 years of teaching experience in several universities and was an associate professor of TEFL.

### Data Analysis

To answer the research questions for the quantitative phase, the researchers inserted the results of the OPT into the SPSS software and employed both descriptive and inferential statistics. As for the qualitative data analysis, the obtained information from the semi-structured interviews was transcribed, coded, and analyzed. A systematic guideline for data coding was adopted from Ary et al. (2014). In the analysis phase, the prominent themes related to learners’ motivation in general and the L2 self-motivating system were identified in particular.

#### Checking the Reliability of Interview

To investigate whether the interview items enjoyed an acceptable level of reliability, the researchers ran inter-coder reliability. The results of this analysis are summarized in Table 2.

**TABLE 2:** Interrater reliability of interview.

Items	Percent agreement (%)	Scott’s Pi	Cohen’s Kappa
Item 1	84	0.84	0.85
Item 2	85	0.85	0.86
Item 3	91	0.91	0.93
Item 4	100	1	1
Item 5	86	0.86	0.87
Item 6	92	0.92	0.93
Item 7	100	1	1

As demonstrated in Table 2, all the items of the interview had an acceptable consistency level. Cohen’s Kappa for these items ranged from 0.84 to 1, which can be considered a high level of consistency.

#### Face Validity

“The extent to which an instrument looks as if it measures what it is intended to measure” (Patton, 1997). Direct measurement of face validity is obtained by asking people to rate the validity of a test as it appears to them. To ensure the face validity of the selected instrument, it was sent to three TEFL experts and professors to review the developed items from different points of view and verify the fact that the instrument was suitable for the intended purposes.

#### Checking the Reliability of Questionnaire

Prior to the administration of the questionnaire for the main study, the researchers piloted it with 30 EFL learners. The obtained data were analyzed using Cronbach’s alpha method to find out the reliability coefficient.

As shown in Table 3, the reliability coefficient of the questionnaire was found to be 0.79 which is considered an acceptable level of reliability.

**TABLE 3:** The reliability index for the motivation questionnaire.

Scale	Number of items	*N*	Cronbach’s alpha	Cronbach’s alpha based on standardized items
Motivation	10	30	0.792	0.791

## Results

### Motivational Factors Influencing EFL Learners English Learning

The participants’ answers to seven interview questions about motivational factors were categorized into nine distinct themes which are presented in Table 4.

**TABLE 4:** Participants’ description of motivational factors in learning English.

Interview	Factors	Themes	Examples
Motivational factors	A. Intrinsic	Personal enjoyment	• Learning English enhances my self-confidence • Mastering English skills gives me a sense of satisfaction and prosperity • I am very interested being able to fluently speak English
		Social prestige	• I want to be recognized and appreciated by others because of my skilled and efficient use of English • I always like to be one step ahead of others
		Being challenged	• I like to engage in challenging tasks that are difficult for a large number of people • I am motivated to work on personally meaningful goals and activities with an optimal level of difficulty
	B. Extrinsic	Academic purposes	• I need to study and understand the contents of academic articles and research papers • I’d like to receive the IELTS, TOEFL, and other international certificates
		Communication	• I like to speak and interact with tourists who travel to my city from foreign countries • I like to be in contact with some native speakers from English-speaking countries through email or other social networks
		Employment	• I want to earn money from teaching and translating English • English helps me to develop and improve my business through being able to study English sources and materials
		Technology	• I learn English to promote and improve my computer knowledge • I learn English to improve my ability to search on-line media and social networks
		Migration	• I like to live in a English-speaking country • I plan emigrate to English-speaking countries and teach English native speakers about the Iranian culture
		Education	• It helps me at university, and if I go abroad to study, I need a language • English knowledge helps me perform better at the university entrance exam (Konkur)

According to Table 4, general themes, i.e., Intrinsic and Extrinsic, related to motivational factors of English learning emerged from the analysis of the participant’s answers to the interview questions. For the first one, three factors were extracted including, (a) personal enjoyment, (b) social prestige and, (c) being challenged. In other words, the participants’ intrinsic motivation and rationale behind learning English were found to be attributed to their interests and love of English, achieving higher social status as well as their tendency to engage in challenging and difficult tasks. For example, concerning personal enjoyment factor, one of the participants said: *“Learning English skills brings about a sense of satisfaction and happiness.”* Concerning being challenged, one of the participants stated: “*I am really motivated to engage in challenging tasks with an optimal level of difficulty.”* Furthermore, concerning the communication theme, another participant answered that: *“I like to get in touch with some native individuals from English-speaking countries through emails or other social networks.”* Regarding pursuing academic purposes, one of the participants asserted that: *“I’d like to attain the highest score possible in IELTS and TOEFL tests and be awarded such important certificates.”* Within the employment theme, another participant responded: *“English helps me to develop and improve my business by being able to understand English sources, texts, and materials.”*

### Motivational Factors Influencing Elementary EFL Learners

The second research question sought to determine the motivational factors which might influence the elementary EFL learners’ tendency to learn English as a foreign language. The elementary level participants’ responses to the interview questions were analyzed and the results of thematic analysis are presented below.

As shown in Table 5, the elementary level EFL learners reported in their responses to the interview questions that both intrinsically and extrinsically-oriented factors can influence their motivation and tendency to engage in learning English as a foreign language. There are two intrinsic factors, i.e., personal enjoyment and social prestige, as the main reasons and rationale behind their efforts and enthusiasm to learn a foreign language. For instance, one of the EFL learners who learned English to gain social prestige asserted that: *“Learning English brings about a kind of high social class and prestige for me in front of my friends and relatives.”* Another participant who learned English for enjoyment and entertainment stated that: “*I am very interested in learning and knowing about a language other than Persian.”*

**TABLE 5:** Motivational factors influencing elementary EFL learners.

Proficiency	Theme	Factors	Example
Elementary	Intrinsic	Personal enjoyment	I am very interested in learning and knowing about a language other than Persian
		Social prestige	Mastery of English brings for me a social class in front of my friends and relatives
		Communication	I’d like to be able to make interactions and establish a relationship with an English native speaker
	Extrinsic	Technology	English learning helps me to work with my PC much more easily and efficiently
		Employment	I’m going to teach English in a language institute

Furthermore, the elementary level participants reported three extrinsic Factors to which their tendency toward learning a foreign language could be attributed. They pointed to the efficient use of new technologies, facilitation of employment, and making effective communications as the extrinsically-oriented factors that encouraged them to learn English. For example, one of the participants reported that: “*Learning English helps me to work with my PC much more easily and efficiently.*” Concerning finding job opportunities, another participant stated that: *“I’m going to be able to teach English one day and make money with that.”* Yet another elementary level EFL learner responded that: *“I’d like to be able to speak fluently with an English native speaker.”*

Most non-proficient and elementary learners who were at the initial stages of EFL learning wanted to learn English based on extrinsic factors especially their needs to find a decent job, to earn money and to interact with individuals who live in an English-speaking country. Moreover, from among these elementary EFL learners, a few considered intrinsic factors as their main source of motivation and primary driving force to learn English.

### Motivational Factors Influencing Intermediate EFL Learners

The second research question also sought to specify the motivational factors which influenced the intermediate EFL learners’ motivation to learn English as a foreign language. The results of a thematic analysis of these learners’ responses to the interview items are illustrated in Table 6.

**TABLE 6:** Motivational factors influencing intermediate EFL learners.

Proficiency	Theme	Factors	Example
Intermediate	Intrinsic	Personal enjoyment	Learning English is an enjoyable and pleasant experience for me
		Communication	Learning English helps me to make effective interactions with tourists who travel to Iran
		Social prestige	When I speak English fluently with a tourist, it makes me feel proud of myself
	Extrinsic	Education	Mastery of English vocabulary and grammar greatly contributes to my success at university entrance exam
		Employment	I’m going to be employed by one of the foreign embassies as either a translator or an interpreter

The information in Table 6 demonstrates that the intermediate EFL Learners considered both intrinsically and extrinsically-oriented factors as the reasons and rationale behind their interest to learn English as a foreign language. This group of EFL learners in their responses to the interview questions declared personal enjoyment and social prestige as the intrinsic factors. In this respect, one of the EFL learners who wanted to learn English to gain social prestige stated that: *“I really like the social class and prestige I gain through learning English and I feel more confident in the community I live in.”* The other participant who learned English to meet personal interests and find enjoyment said that: “*When I speak English fluently with a tourist, it makes me proud of myself.”* Concerning the extrinsic factors, the intermediate EFL learners reported three extrinsic factors which affected their motivation to learn English. To the intermediates, educational needs, employment opportunities, and making communication were the major driving forces to engage in learning English. For example, one of the participants responded that: “*Mastery of English vocabulary and grammar would greatly contribute to my likely success in the university entrance exam*.” With regard to finding a decent job, another respondent stated that: *“I’m going to be able to teach English in a private English language institute.”* With regard to communicating with English native speakers, a participant said that: *“I’d like to be able to communicate well with an English native speaker.”*

Most intermediate English learners who were considered to possess a relatively good command of English language skills and proficiency were more extrinsically-motivated. Finding a decent job, pursuing academic careers, and communicating well with native speakers were among such motivational factors. Moreover a few intermediate language learners regarded intrinsic factors as the primary source of motivation and tendency to learn English.

### Motivational Factors Influencing Advanced EFL Learners

The last part of the second research question intended to explore the motivational factors which underpinned the advanced EFL learners’ disposition to pursue learning of English at higher levels of language proficiency. This group of the participant’s responses to the interview questions were analyzed and the results of thematic analysis are demonstrated in Table 7.

**TABLE 7:** Motivational factors influencing advanced EFL learners.

Proficiency	Theme	Factors	Example
Advanced	Intrinsic	Personal enjoyment	Learning different aspects of a foreign language is a deeply enjoyable and pleasant experience for me
	Extrinsic	Academic purposes	I’d like to be able to get as many international English certificates as possible
		Employment	I’m going to be qualified as an employee in a foreign company and organization
		Migration	I’d like to get admission to a reputable university in Canada and emigrate to this foreign country

As demonstrated in Table 7, the advanced level EFL learners declared in their responses to the interview questions that both intrinsically and extrinsically-oriented factors can influence their tendency to pursue the path of learning English as a foreign language. They put the focus on only one intrinsic factor, i.e., personal enjoyment, as the main internal desire which could justify their efforts to learn a foreign language much more efficiently. For instance, one of the EFL learners who considered English learning as an enjoyable and pleasant learning experience said that: *“Learning different aspects of a foreign language is a deeply enjoyable and pleasant experience for me.”* However, this group of participants reported three extrinsically-oriented factors to which their desire to learn English could be attributable. They introduced and recognized the facilitation of fulfilling academic purposes, provision of good employments; and migration opportunities as the extrinsically-oriented motivations which might direct and encourage them to improve their English proficiency level as much as possible. For example, one of the respondents said that: “*I’d like to receive as many English learning and teaching certificates as possible.*” Another one contended that: *“I’d like to be admitted to a reputable university in Canada and move to this foreign country.”* Accordingly, it can be said that most advanced EFL learners reported such extrinsic motivating factors as pursuing academic careers, finding job opportunities, and migrating to an English-speaking country as the secondary driving forces to learn English.

### The Common Motivational Factors Among the Participants

The third research question aimed at examining the motivational factors underlying the participants’ tendency to learn English which was common among the three groups of EFL learners in terms of their proficiency level. In all three groups of EFL learners, i.e., elementary, intermediate, and advanced, both intrinsically and extrinsically-oriented factors were found to play an effective role in fostering their tendencies toward English learning. More specifically, the analysis of their responses to the interview questions indicated some similarities and differences. Concerning the intrinsic factors, the “personal enjoyment” was reported by all three groups of the participants as one of main rationale behind English learning. In the same vein, social prestige was also found to be a common factor that might provide ground and motivation to learn English among elementary and intermediate EFL learners. On the other hand, concerning the extrinsically-oriented factors, finding job opportunities was found to be a common factor which the participants across all three groups of language proficiency expressed as their driving force to learn English. More precisely, when comparing the elementary and intermediate EFL learners, not only were finding job opportunities reported by both beginner and intermediate language learners as one main driving force to pursue learning English, commutating well with native speakers was also another motivating factor that were alluded to by both groups of language learners. In other words, elementary and intermediate participants had unanimously agreed upon these two extrinsic factors. Table 8 demonstrates the above-mentioned result.

**TABLE 8:** Common motivational factors among the three group of participants.

	Motivational factors	Proficiency level		
		Elementary	Intermediate	Advanced
Extrinsic	Employment	+	+	+
Intrinsic	Social prestige	+	+	
	Communication	+	+	
	Personal enjoyment	+	+	+

To sum up, it was found that personal enjoyment and finding decent job opportunities were the two motivational factors which all the participants, regardless of their English proficiency level, declared as the major driving forces to pursue their English learning.

### Quantitative Data Analyses Results

To further investigate the motivational factors and their differences in terms of participants’ English proficiency level, the obtained data from the motivational factor questionnaire was analyzed and used to answer the last research question of the present study quantitatively.

#### Comparing the Participants Motivational Factors in Terms of Language Proficiency

The fourth research question of this study intended to investigate quantitatively whether the participants in three proficiency groups (i.e., elementary, intermediate, and advanced) were reported to be motivated by different factors in learning English. To answer this research question, a one-way ANOVA analysis was conducted to draw a comparison among the three groups. The descriptive statistics of the participants’ self-perceived motivational factors are summarized in Table 9.

**TABLE 9:** Descriptive statistics of the participants’ self-perceived motivational factors in terms of English proficiency.

Self-perceived motivational factors	Proficiency level	*N*	Mean	SD
	Elementary	82	4.49	0.98
	Intermediate	70	4.23	0.87
	Advanced	28	3.24	0.56

As indicated in Table 9, the highest mean score belongs to the elementary level respondents (*M* = 4.49, SD = 0.98) and the lowest mean score (*M* = 3.24, SD = 0.56) to the advanced level participants. To ensure that the differences are statistically significant the data in Table 10 should be taken into account.

**TABLE 10:** ANOVA analysis for comparing mean scores in respondents’ motivational factors.

	Sum of squares	Df	Mean square	*F*	Sig.
Between groups	234.123	2	154.547	38.217	0.001
Within groups	98.021	67	3.468		
Total	221.12	70			

There was a statistically significant difference at the *p* < 0.05 level in driving motivational factors to English learning for the three groups: *F* (2, 67) = 38.21, *p* = 0.001. In addition to reaching statistical significance, the actual difference in mean scores between the groups was also large. The effect size, calculated using eta squared, was 0.75. As displayed in Table 11, *post hoc* comparisons using the Scheffe test indicated that the mean score for Group 1 (*M* = 15.66, SD = 0.9861) was not significantly different from Group 2 (*M* = 4.23, SD = 0.8763). While, it was found that Group 3 (*M* = 3.24, SD = 0.5697) did differ significantly from both Groups 1 and 2. In other words, the findings indicated that the EFL elementary English learners were not significantly different from the intermediate English learners in terms of the driving forces that pushed them forward to learn English.

**TABLE 11:** Multiple comparison of groups.

(I) Id	(J) Id	Mean difference	Sig.	95% confidence interval	
				Lower bound	Upper bound
(1) Intermediate	2	0.30	0.123	0.000	–10.771
(2) Upper-intermediate	3	–0.99	0.003	0.000	–8.843
(3) Advanced	1	–1.25	0.001	0.000	–13.07

## Discussion

This study was set to delve into the motivational factors that affected Iranian EFL leaners across differing levels of language proficiency. To pursue this line of inquiry, a concurrent triangulation mixed-methods design was adopted in which both quantitative and qualitative data were concurrently collected to provide thorough responses to the research questions.

The first obtained results from the qualitative parts of the study suggested that for all the participants the motivational factors which might encourage and direct the EFL learners to learn English can stem from either intrinsic or extrinsic tendencies and orientations. Advanced learners were reported to resort to intrinsic factors as the driving forces for learning English. They declared some factors such as personal enjoyment and being challenged as the reasons for which they would like to pursue their English learning process. Beginners and intermediate language learners expressed the extrinsically-oriented factors as their source of motivation toward English learning and pointed to reasons such as getting a job, pursuing education, earning more money, traveling to foreign countries, fulfilling academic purposes, making efficient use of technology, as well as making effective interactions with native speakers as the motivational factors which might encourage them to learn English. However, majority of elementary and intermediate English language learners were more extrinsically-motivated to learn English and their underlying reasons to pursue English were primarily extrinsically-oriented.

The second major qualitative finding of this study suggested that many novice English learners tended to learn English based on extrinsic factors, especially employment and communication requirements. Moreover, the obtained results from qualitative analysis indicated that the participants, regardless of their proficiency level, unanimously considered personal enjoyment (as an intrinsic factor); and getting employment opportunities (as an extrinsic one) as the two significant factors which underlay their motivation to learn English.

The qualitative finding in this section seems to be partly in agreement with that of Nakamura (2019) who reported that English is regarded as an essential means of global communication and a tool to provide access to information needed for technical and scientific assistance in this globalized digital world. Furthermore, the results concur with that of Wang et al. (2021) that indicated drawing on an individual’s positive emotions like motivation can greatly enhance learning quality and lead to L2 development and progress. The results of the quantitative part are also consistent with that of Wallace and Leong (2020) who delved into the motivational factors that affected young EFL learners and found that majority of learners were highly motivated and learned English for both integrative and instrumental purposes. Furthermore, the findings of present study are congruent with that of Abdullah et al. (2019) who examined the effect of carrying out the FCM on EFL learners’ motivation with a mixed-methods approach and found that FCM contributed greatly to increasing EFL learners’ motivation over time.

To further investigate the qualitatively identified differences between the three groups of participants (i.e., elementary, intermediate, and advanced) in terms of the driving motivational forces to learn English, a quantitative analysis was also conducted using ANOVA statistics. It was found that despite the reported distinctions in the qualitative results, in the quantitative analysis, there was only a significant difference between the advanced EFL learners and their elementary and intermediate counterparts with regard to the motivational factors affecting their English learning. Such motivational differences across varying levels of language proficiency can be attributed to the purposes, perspectives, environment, and family class. More precisely, the educational and schooling system in Iran necessitates that both teachers and learners to resort and being largely accustomed to largely to the traditional procedures of language teaching, testing, and learning in which the main focus is on the prescribed and passive teaching and learning materials. The students in this learning atmosphere are not expected to show enough motivation, self-confidence, and interest to engage in innovative learning activities in the classroom and obtain desired goals and objectives. A great number of families even teachers and students are not satisfied with the achievements of English learning. The low quality and ineffectiveness of the schooling system seem to be among the most crucial factors that exacerbates the this dissatisfaction.

On the other hand, the commonality of employment and personal enjoyment as motivational factors among Iranian learner’s English learning could be considered an expected result which might not come as a surprise to some extent. Iran’s increasingly tight job market, as a result of poor economic policies, as well as global sanctions turn employment into a big challenge for young people. Therefore, one of the most prevalent driving forces among the Iranians, specially the young educated ones to pursue an academic career is to get a decent job and be given a good and proper job opportunity. Learning a foreign language like English is no exception.

## Conclusion

In this study, a mixed-methods design with a concurrent approach was adopted to explore and compare the categories of motivational factors which foreground the EFL learners’ tendencies toward learning English as a foreign language. The obtained data from a motivation questionnaire and interview questions were analyzed using some inferential statistics and thematic analysis. It was found that the motivational factors which direct the EFL learners to English learning can result from either intrinsic or extrinsic tendencies. The recognized intrinsically-based motivational factors were personal enjoyment and being challenged which were considered as the reasons for which they would like to pursue their English learning process. Beginner and intermediate language learners expressed the extrinsically-oriented factors as their source of motivation toward English learning proposed reasons such as getting a job, pursuing education, earning more money, traveling to foreign countries, pursuing academic career, making efficient use of technology, and effectively interacting with native speakers as the motivational factors which might motivate them to learn English. Furthermore, it was concluded that a large number of novice English learners tend to learn English based on extrinsic factors, especially employment and communication requirements. Intermediate EFL learners’ tendency to learn English could be attributed to being successful and getting the desired achievement in education, providing a lot of employment opportunities, and making effective interactions with native speakers. Furthermore, most of the advanced learners considered academic purposes, employment, and migration as the main reasons which might underpin their tendency to maintain learning English.

Moreover, educators should be more cautious in choosing the right instructional materials and pedagogical tools. To decrease demotivation, teachers are recommended to pay due attention to the proper selection and adoption of teaching methodology and their manner that can boost learners’ motivation. Furthermore, teachers need to integrate all language skills, instead of vocabulary and grammatical rules at the expense of oral skills. To motivate language learners, teachers need to take into account the contextual elements and learners’ personal experiences as well.

In the same vein, materials developers are highly recommended to revise instructional materials to increase students’ motivation. Learners’ negative perceptions about and attitudes toward the second language could be changed by promoting the content of textbooks. English textbooks might fail to attract the learners’ interests and decrease their motivation by putting too much emphasis on grammar and vocabulary. Instead, textbooks should provide opportunities for classroom interaction and enable educators to improve learners’ overall oral skills. Accordingly, materials developers need to develop intriguing tasks and topics for the texts in their textbooks to promote learners’ motivation. Regarding students’ negative beliefs and attitudes toward the English speaking community as a demotivating factor in learning English, learners’ motivation can be readily boosted through incorporating cultural information in students’ English textbooks. For instance, cultural tasks can be appropriately included in the language lessons to improve the quality of instructional materials. Educators and instructors need to play their pivotal parts as motivators to students who are struggling to learn a second or foreign language.

In the Iranian educational system, the English curriculum often focuses on preparing L2 learners to pass their exams. English instructors can pay more attention to the communicative facets of the English language in their instructional practices. In this regard, English teachers can maintain and enhance learners’ L2 motivation through various activities in such pedagogic books. This study might provide practitioners and educators with some useful insights into English planning and policy, and it would also offer suggestions for English syllabus designers and material developers in Iran that are congruent with L2 learners’ motivational orientations. Finally, this study might help enhance the organization and presentation of current English-language academic sources in various pedagogic settings.

Nevertheless, some limitations should be taken into account, and caution should be exercised in interpreting the findings of the qualitative part of study because of the following reasons. First, generalizability of the findings could be undermined due to the included sample in the qualitative part. Second, because of the difficulty of unraveling and coming up with the most typical motivational themes across varying language proficiency levels and the relative subjectivity inherent in such thematic analysis, replication research is also required to corroborate the results of the present study. Finally, EFL learners might have suffered from a self-flattery syndrome and overrated themselves to project an ideal self-image of themselves when answering the items of the motivation questionnaire.

The effect of adopting motivational strategies on the EFL learners’ attitudes toward foreign language learning can be investigated. Second, the effectiveness of intrinsic versus extrinsic motivation strategies on language learning achievement can be explored among EFL students at tertiary level. Third, this line of inquiry can be pursued by investigating the correlation between extrinsic and extrinsic motivation with such variables as self-efficacy and anxiety. Finally, motivation is just one of the several elements that could affect the learning process and outcomes. Therefore, instructors need to closely attend to other pertinent factors such as students’ cognitive style, personality factors and language aptitude to further engage them in the learning process. The motivational factors discussed earlier need to be complemented by further empirical research as well.

## Data Availability Statement

The original contributions presented in the study are included in the article/supplementary material, further inquiries can be directed to the corresponding author/s.

## Ethics Statement

Ethical review and approval was not required for the study on human participants in accordance with the local legislation and institutional requirements. Written informed consent from the participants’ legal guardian/next of kin was not required to participate in this study in accordance with the national legislation and the institutional requirements.

## Author Contributions

RB was the corresponding author and contributed more than AF. RB as the corresponding author was the main contributor of this research paper. Both authors contributed to the article and approved the submitted version.

## Conflict of Interest

The authors declare that the research was conducted in the absence of any commercial or financial relationships that could be construed as a potential conflict of interest.

## Publisher’s Note

All claims expressed in this article are solely those of the authors and do not necessarily represent those of their affiliated organizations, or those of the publisher, the editors and the reviewers. Any product that may be evaluated in this article, or claim that may be made by its manufacturer, is not guaranteed or endorsed by the publisher.
